# Development and validation of a clinical score to estimate progression to severe or critical state in COVID-19 pneumonia hospitalized patients

**DOI:** 10.1038/s41598-020-75651-z

**Published:** 2020-11-13

**Authors:** Francisco Gude, Vanessa Riveiro, Nuria Rodríguez-Núñez, Jorge Ricoy, Óscar Lado-Baleato, Tamara Lourido, Carlos Rábade, Adriana Lama, Ana Casal, Romina Abelleira-París, Lucía Ferreiro, Juan Suárez-Antelo, María E. Toubes, Cristina Pou, Manuel Taboada-Muñiz, Felipe Calle-Velles, Plácido Mayán-Conesa, María L. Pérez del Molino, Cristóbal Galbán-Rodríguez, Julián Álvarez-Escudero, Carmen Beceiro-Abad, Sonia Molinos-Castro, Néstor Agra-Vázquez, María Pazo-Núñez, Emilio Páez-Guillán, Pablo Varela-García, Carmen Martínez-Rey, Hadrián Pernas-Pardavila, María J. Domínguez-Santalla, Martín Vidal-Vázquez, Ana T. Marques-Afonso, Arturo González-Quintela, José R. González-Juanatey, Antonio Pose, Luis Valdés

**Affiliations:** 1 Research Methods Group, Epidemiology Department, Health Research Institute of Santiago de Compostela (IDIS), University Clinical Hospital of Santiago, Santiago de Compostela, Spain; 2 Pulmonology Department, University Clinical Hospital of Santiago, Santiago de Compostela, Spain; 3 Department of Statistics, Mathematical Analysis, and Optimization, University of Santiago de Compostela, Santiago de Compostela, Spain; 4 Pulmonology Department, Interdisciplinary Group of Research in Pulmonology, University Clinical Hospital of Santiago, Health Research Institute of Santiago de Compostela (IDIS), Santiago de Compostela, Spain; 5 Anesthesiology Department, University Clinical Hospital of Santiago, Santiago de Compostela, Spain; 6 Primary Health Care, University Clinical Hospital of Santiago, Santiago de Compostela, Spain; 7 Emergency Department, University Clinical Hospital of Santiago, Santiago de Compostela, Spain; 8 Microbiology Department, University Clinical Hospital of Santiago, Santiago de Compostela, Spain; 9 Intensive Care Unit, University Clinical Hospital of Santiago, Santiago de Compostela, Spain; 10 Internal Medicine Department, University Clinical Hospital of Santiago, Santiago de Compostela, Spain; 11 Cardiology Department, CIBERCV, Health Research Institute (IDIS), University Clinical Hospital of Santiago, Santiago de Compostela, Spain

**Keywords:** Diseases, Medical research

## Abstract

The prognosis of a patient with COVID-19 pneumonia is uncertain. Our objective was to establish a predictive model of disease progression to facilitate early decision-making. A retrospective study was performed of patients admitted with COVID-19 pneumonia, classified as severe (admission to the intensive care unit, mechanic invasive ventilation, or death) or non-severe. A predictive model based on clinical, laboratory, and radiological parameters was built. The probability of progression to severe disease was estimated by logistic regression analysis. Calibration and discrimination (receiver operating characteristics curves and AUC) were assessed to determine model performance. During the study period 1152 patients presented with SARS-CoV-2 infection, of whom 229 (19.9%) were admitted for pneumonia. During hospitalization, 51 (22.3%) progressed to severe disease, of whom 26 required ICU care (11.4); 17 (7.4%) underwent invasive mechanical ventilation, and 32 (14%) died of any cause. Five predictors determined within 24 h of admission were identified: Diabetes, Age, Lymphocyte count, SaO_2_, and pH (DALSH score). The prediction model showed a good clinical performance, including discrimination (AUC 0.87 CI 0.81, 0.92) and calibration (Brier score = 0.11). In total, 0%, 12%, and 50% of patients with severity risk scores ≤ 5%, 6–25%, and > 25% exhibited disease progression, respectively. A risk score based on five factors predicts disease progression and facilitates early decision-making according to prognosis.

## Introduction

The first cases of pneumonia of unknown origin were detected in Wuhan (Hubei, China) in early December 2019^1^. On 7 January 2020, Chinese scientists isolated a novel coronavirus that was named as severe acute respiratory syndrome coronavirus 2 (SARS-CoV-2), and the relevant disease was called coronavirus-2019 disease (COVID-19)^2^. Since then, the dramatic increase in cases has posed numerous challenges to even the most sophisticated and advanced health systems, which led the World Health Organization (WHO) to declare the outbreak a pandemic in March 2020^3^. To date, health systems worldwide have experienced an exponential increase in hospitalizations and admissions to intensive care units (ICUs) associated with COVID-19^4^.

COVID-19 can cause a wide variety of symptoms ranging from asymptomatic infection to life-threatening complications such as acute respiratory distress, multi-organic failure, and death^1,5–7^. Some studies have evidenced that older patients with comorbidities (including arterial hypertension, cardio-respiratory disease or diabetes)^6^ and patients with more elevated levels of cytokines in blood^1^ are the ones at a higher risk for experiencing severe complications^8,9^.

At this time, there are no specific vaccines or treatments for COVID-19. Accurate diagnosis and prognosis of the disease are crucial to alleviating the burden on the health system while the best care possible is provided to patients. A predictive model that combines several variables or parameters to estimate the risk for a poor outcome would help the clinician to estimate the prognosis of patients when limited healthcare resources are available. Thus, early identification of patients at risk of serious complications is clinically relevant^10^. A recent systematic literature review found ten prognostic models for predicting mortality or progression to severe disease, but only a study involved patients from countries other than China, and all studies had been categorized as being at a high risk of bias^11^.

Therefore, the aim of this study was to develop and validate a prognostic model to identify inpatients with COVID-19 pneumonia at a greater risk for developing severe/critical complications, including death.

## Methods

### Source of data

Data were collected from the medical reports of patients diagnosed with COVID-19 and admitted to the Complejo Hospitalario Universitario of Santiago de Compostela in Spain, a hospital with over 1000 beds, from March 12, 2020 (date of first COVID-19 diagnosis) to April 11. The study was conducted in accordance with the guidelines of the Declaration of Helsinki and the principles of good clinical practice and was approved by the Institutional Review Board (IRB) of the Galician Health Service on April 3, 2020 (#2020/194). Informed consent forms were waived by the IRB.

A confirmed case of COVID-19 was defined as a positive result in the reverse transcription polymerase chain reaction (RT-PCR) test on samples obtained from nasal or throat swabs performed in accordance with WHO protocol^12^. Only laboratory-confirmed cases were considered for analysis. Patients with uncomplicated disease, with symptoms of upper airway infection, headache, myalgia, anosmia, dysgeusia or anorexia, but with an SaO_2_ > 95% and a respiratory rate < 25 breaths/min, were monitored as follows: (1) At home by the TELEA system^13^, a home monitoring platform that allows to monitor respiratory and heart rate, temperature, and SaO_2_; (2) patients without an Internet connection at home were monitored via 2–3 telephone calls daily. If the clinical status of the patient deteriorated, a physician contacted them to decide where hospitalization was required or not; (3) previously-institutionalized patients or those without enough assistance at home were transferred to a socio-health center adapted as a hospital. All patients diagnosed with COVID-19 pneumonia were hospitalized.

All patients with COVID-19 pneumonia were eligible for inclusion. Pneumonia was defined as an acute respiratory disorder characterized by cough, chest radiograph findings of lung shadowing that is likely to be new, and fever ≥ 4 days or dyspnea/tachypnea^14^. Exclusion criteria were simultaneous infection by another germ or in other organ. Fever was defined as an axillary temperature ≥ 37.5 °C.

The data extracted from the medical history of patients included symptoms, clinical signs, and laboratory test and radiological results at admission (+ 1 day). The comorbidities considered were arterial hypertension, diabetes mellitus, chronic obstructive pulmonary disease, other respiratory diseases, kidney and liver disease, heart failure, ischemic heart disease, heart valve surgery, active neoplasm, systemic disease, and psoriasis. Previous use of drugs such as angiotensin-converting enzyme inhibitors, angiotensin II receptor antagonists, statins, corticosteroids, immunosuppressants, antiplatelet agents, and anticoagulants were recorded. The totality of laboratory tests were performed as a function of the clinical care needs of the patients. Determinations in blood included a complete hemogram, coagulation tests (including D-dimer), an evaluation of liver and kidney function, and determination of electrolytes, C-reactive protein (CRP), procalcitonin, lactate dehydrogenase (LDH), creatine kinase, ferritin, and interleukin 6. COVID-19 was considered severe and the patient was candidate to ICU admission if required mechanical ventilation or had a fraction of inspired oxygen (FiO_2_) of at least 60% or more^15^. Radiological anomalies were collected from reports of the Unit of Radiology.

Outcomes of interest were death from any cause, use of mechanic invasive ventilation, or ICU stay.

### Statistical analysis

Multiple imputation was used to impute missing data by creating 100 datasets. Missing values were predicted on the basis of all other predictors considered for assessing outcomes^16^. For each of them, 250 bootstrap datasets were generated, and backwards feature selection with the Akaike Information Criterion was performed on every set^17^.

The predictors returned by this procedure in 70% of datasets were selected for the final model, and their regression coefficients were calculated according to “Rubin's Rules”^18^. We also studied the possible nonlinear effect of each predictor on the outcome by means of Generalized Additive models (GAM) using splines^17^. Results are presented as Odds Ratio (OR) with 95% confidence intervals (CI). The Nagelkerke R^2^ was used to calculate the proportion of variance in clinical outcomes that could be explained by the selected predictors. The different aspects of model performance were studied, including calibration and discrimination^19^. Calibration was assessed using the Brier score, and by plotting the non-parametric estimate of the association between the observed frequencies and the predicted probabilities. The receiver operating characteristics (ROC) curves (and the correspondent area under the ROC curve-AUC) were calculated to test for discrimination. To correct optimism, internal validation was performed using the bootstrap procedure. The procedure was repeated in each imputed dataset, and the average estimates for the AUC, the Brier Score, and the Nagelkerke R^2^ were extracted to assess discrimination, calibration, and overall fit, respectively^20^. The final model was selected to derive a score for clinical use and a nomogram was created. Criteria for this selection included both discriminant ability (defined by the AUC) and model simplicity. Statistical significance was set at p < 0.05. All statistical analyses were carried out in R version 3.5.1 using the mice rms and psfmi packages). These packages are freely available at https://cran.r-project.org. The analysis conforms to TRIPOD reporting standards^21^.

## Results

**Figure 1:**
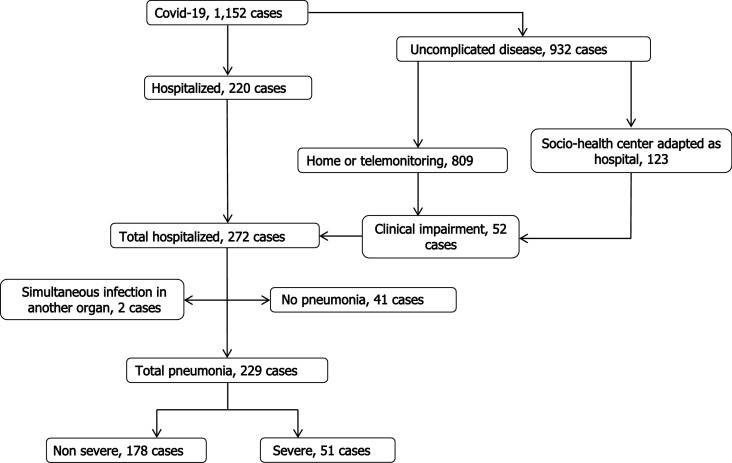
Flow chart for management of patients with Covid-2019 in health area of Santiago de Compostela.

During the study period, 1152 patients were infected by COVID-19, of whom 229 (19.9%) were admitted for pneumonia. None of the hospitalized patients were lost to follow-up. During hospitalization, 51 (22.3%) cases progressed to severe disease (Fig. 1), of whom 26 (11.4%) needed intensive care, 17 (7.4%) underwent invasive mechanical ventilation, and 32 (14%) died of any cause. The majority of patients (90%) received antibiotic therapy and hydroxychloroquine, and 83.8% received lopinavir/ritonavir. Additionally, 30% were given systemic glucocorticoids, and 8.3% were administered tocilizumab.

### Clinical characteristics

Baseline demographic, clinical, and laboratory data are presented in Table 1. The most common symptoms at onset of illness were cough (77.3%), fever (75.5%), and dyspnea (52.8%). Patients with severe disease were significantly older than those with nonsevere disease and were more likely to have higher systolic blood pressure levels (p = 0.003), and lower SaO_2_ concentrations (p < 0.001). The presence of comorbidities such as diabetes, heart failure, and chronic kidney disease was significantly higher in patients who progressed to severe disease. No statistically significant differences were observed between the two groups in main symptoms, although slightly more patients in the severe group manifested confusion (p = 0.025).

**Table 1:** Clinical characteristics of the study patients.

	All patients		Severe course		Odds ratio (95% CI)	*p* value
	Missing	(n = 229)	Yes (n = 51)	No (n = 178)		
**Age, yr**	0	68 (56, 75)	74 (68, 83)	65 (55, 73)	1.06 (1.03, 1.09)	< 0.001
**Male sex**	0	139 (60.7)	40 (78.4)	99 (55.6)	2.90 (1.40, 6.02)	0.004
**Temperature ≥ 37.5 °C**	1	82 (36.0)	23 (45.1)	59 (33.3)	1.64 (0.87, 3.10)	0.125
**Systolic blood pressure, mmHg**	0	130 (118, 140)	134 (120, 151)	129 (118, 139)	1.02 (1.01, 1.04)	0.003
**Diastolic blood pressure, mmHg**	0	74 (66, 80)	75 (66, 82)	74 (66, 80)	1.01 (0.98, 1.04)	0.521
**Heart rate, beats/min**	1	83 (74, 94)	86 (74, 96)	81 (75, 91)	1.01 (0.99, 1.04)	0.246
**SaO**_**2**_	8	94 (92, 96)	92 (88, 95)	95 (93, 96)	0.87 (0.81, 0.93)	< 0.001
**Symptoms**						
Fever	0	177 (77.3)	40 (78.4)	137 (77.0)	1.09 (0.51, 2.31)	0.826
Cough	0	173 (75.5)	37 (72.5)	136 (76.4)	0.82 (0.40, 1.65)	0.573
Shortness of breath	0	121 (52.8)	34 (66.7)	87 (48.9)	2.09 (1.09, 4.02)	0.027
Thoracic pain	0	21 (9.2)	2 (3.9)	19 (10.7)	0.34 (0.08, 1.52)	0.158
Diarrhea	0	57 (24.9)	10 (19.6)	47 (26.4)	0.68 (0.32, 1.46)	0.324
Anosmia	6	17 (7.4)	2 (4.0)	15 (8.7)	0.44 (0.10, 1.99)	0.285
Dysgeusia	7	25 (10.9)	3 (6.0)	22 (12.8)	0.44 (0.12, 1.52)	0.192
Confusion	0	9 (3.9)	5 (9.8)	4 (2.2)	4.73 (1.22, 18.3)	0.025
**Treatment**						
Angiotensin-converting enzyme inhibitors	0	21 (9.2)	4 (7.8)	17 (9.6)	0.81 (0.26, 2.51)	0.710
Angiotensin II receptor antagonists	0	55 (24.0)	15 (29.4)	40 (22.5)	1.44 (0.72, 2.89)	0.308
Statins	0	92 (40.2)	23 (45.1)	69 (38.8)	1.30 (0.69, 2.43)	0.417
Corticosteroids	0	19 (8.3)	3 (5.9)	16 (9.0)	0.63 (0.18, 2.26)	0.482
Immunosupressors	0	15 (9.6)	1 (2.0)	14 (7.9)	0.23 (0.03, 1.86)	0.166
Anticoagulants	0	20 (8.7)	13 (5.5)	7 (3.9)	8.36 (3.12, 22.3)	< 0.001
Antiplatelet agents	0	28 (12.2)	5 (9.8)	23 (12.9)	0.73 (0.26, 2.03)	0.550
**Medical history**						
Chronic obstructive pulmonary disease	0	17 (7.4)	7 (13.7)	10 (5.6)	2.67 (0.96, 7.42)	0.059
Arterial hypertension	0	101 (44.1)	27 (52.9)	74 (41.6)	1.58 (0.85, 2.96)	0.151
Diabetes mellitus	0	50 (21.8)	24 (47.1)	26 (14.6)	5.20 (2.61, 10.3)	< 0.001
Chronic renal disease	0	21 (9.2)	9 (17.6)	12 (6.7)	2.96 (1.17, 7.50)	0.022
Coronary heart disease	0	17 (7.4)	5 (9.8)	12 (6.7)	1.50 (0.50, 4.49)	0.465
Heart failure	0	14 (6.1)	10 (19.6)	4 (2.2)	10.6 (3.25, 35.5)	< 0.001
Cancer	0	12 (5.2)	5 (9.8)	7 (3.9)	2.66 (0.81, 8.75)	0.109
Systemic disease	0	19 (8.3)	2 (3.9)	17 (9.6)	0.39 (0.09, 1.73)	0.214
Pulmonary disease	1	34 (14.8)	10 (19.6)	24 (13.6)	1.55 (0.69, 3.51)	0.288

Data are n (%). 95%CI, 95% Confidence Interval. Severe course was death from any cause, use of mechanic invasive ventilation, or intensive care unit stay.

On admission, patients who progressed to severe disease had a lower baseline lymphocyte and platelet count, lower levels of hemoglobin, and higher levels of neutrophils, serum creatinine, urea, CRP, and interleukin-6 (all p < 0.01) (Table 2). Abnormalities on chest X-ray images were detected in all patients. More than half of patients (57.2%) had bilateral pneumonia (Table 3). The most common findings in 51 patients who progressed to severe disease were bilateral multiple areas of consolidation (28; 54.9%) (Table 3). Table 3 shows a comparison of gasometric parameters between the two groups by level of severity.

**Table 2:** Laboratory characteristics of the patients hospitalized with COVID-19 pneumonia at admission.

Characteristics	All patients		Severe course		Odds ratio (95% CI)	*p* value
	Missing	(n = 229)	Yes (n = 51)	No (n = 178)		
White-cell count, 10^2^ cells/mm^3^	8	57.1 (45.1, 72.4)	69.7 (51.3, 94.0)	54.4 (44.2, 67.9)	1.02 (1.01, 1.03)	0.002
Lymphocyte count, 10^2^ cells/mm^3^	8	10.1 (6.6, 14.3)	6.6 (4.6, 8.7)	11.3 (7.7, 15.0)	0.84 (0.77, 0.91)	< 0.001
Neutrophil count, 10^2^ cells/mm^3^	8	38.8 (29.3, 55.4)	57.5 (38.3, 76.3)	36.7 (27.8, 50.0)	1.03 (1.01, 1.04)	< 0.001
Platelet count, 10^3^ cells/mm^3^	8	213 (158, 285)	183 (132, 227)	227 (173, 291)	1.00 (0.99, 1.00)	0.028
Haemoglobin, g/dL	9	13.1 (12.1, 14.0)	12.6 (10.6, 13.8)	13.3 (12.5, 14.0)	0.73 (0.60, 0.89)	0.002
C-reactive protein, mg/dL	14	6.8 (3.2, 12.8)	11.7 (5.9, 17.4)	6.6 (2.9, 11.3)	1.12 (1.06, 1.18)	< 0.001
Procalcitonin, ng/mL	13	0.12 (0.07, 0.23)	0.2 (0.1, 0.8)	0.1 (0.1, 0.2)	1.11 (0.91, 1.36)	0.313
Lactate dehydrogenase, U/L	17	472 (367, 631)	613 (467, 736)	460 (374, 589)	1.23 (1.05, 1.44)	0.012
Aspartate aminotransferase, UI/L	14	32 (24, 47)	36 (27, 52)	31 (23, 45)	1.01 (1.00, 1.02)	0.135
Alanine aminotransferase, UI/L	10	28 (20, 48)	27 (20, 43)	29 (20, 49)	1.00 (0.98, 1.01)	0.479
Gamma-glutamil transferase, UI/L	9	34 (22, 61)	45 (25, 77)	34 (22, 58)	1.03 (0.79, 1.33)	0.839
Total bilirubin, mg/dL	10	0.6 (0.5, 0.9)	0.5 (0.4, 0.8)	0.7 (0.5, 0.9)	0.50 (0.19, 1.29)	0.151
Creatine kinase, UI/L	31	76 (48, 140)	129 (84, 253)	66 (45, 107)	1.28 (1.05, 1.56)	0.016
Creatinine, mg/dL	7	0.8 (0.6, 1.0)	0.9 (0.7, 1.4)	0.8 (0.7, 1.0)	2.23 (1.24, 4.01)	0.007
Urea, mg/dL	7	39 (30, 54)	59 (33, 82)	38 (30, 48)	1.02 (1.01, 1.03)	< 0.001
D-dimer, ng/mL	13	671 (414, 1118)	919 (672, 1550)	610 (401, 1049)	1.02 (1.00, 1.04)	0.061
Troponin, ng/mL	37	0.02 (0.02, 0.02)	0.02 (0.02, 0.11)	0.02 (0.02, 0.02)	1.10 (0.95, 1.28)	0.184
Prothrombin time, seg	9	12.5 (11.7, 13.4)	12.9 (12.0, 14.1)	12.5 (11.7, 13.3)	1.11 (1.00, 1.23)	0.054
APTT, seg	9	30.6 (28.0, 32.8)	31.7 (28.0, 35.2)	30.6 (28.1, 32.6)	1.03 (0.99, 1.08)	0.162
Interleukin-6, pg/mL	62	26.8 (13.5, 57.0)	78.4 (42.8, 121.2)	20.9 (11.4, 40.7)	1.01 (1.01, 1.02)	< 0.001

Data are median (IQR). 95%CI, 95% Confidence Interval; APTT, activated partial thromboplastin time.

Severe course was death from any cause, use of mechanic invasive ventilation, or intensive care unit stay.

**Table 3:** Radiological and gasometric characteristics of patients hospitalized with COVID-19 pneumonia at admission.

	All patients		Severe course		Odds ratio (95%CI)	*p* value
	Missing	(n = 229)	Yes (n = 51)	No (n = 178)		
**Radiologic**	0					
Unilateral consolidation		98 (42.8)	12 (23.5)	86 (48.3)	Reference	–
Bilateral consolidation		81 (35.4)	28 (54.9)	53 (29.8)	3.7 (1.7, 8.1)	0.005
Bilateral interstitial abnormalities		26 (11.3)	5 (9.8)	21 (11.8)	1.7 (0.5, 5.4)	0.361
Bilateral consolidation + interstitial		24 (10.5)	6 (11.8)	18 (10.1)	2.4 (0.8, 7.2)	0.122
**Gasometry**						
FiO_2_	21	0.21 (0.21, 0.21)	0.21 (0.21, 0.21)	0.21 (0.21, 0.21)	50 (0.2, 12,900)	0.166
pH	25	7.46 (7.13, 7.48)	7.47 (7.41, 7.49)	7.46 (7.43, 7.48)	0.57 (0.00, 680)	0.877
PaCO_2_, mm Hg	25	32.6 (29.4, 35.7)	31.3 (28.5, 36.0)	32.8 (30.0, 35.6)	0.97 (0.91, 1.03)	0.347
PaO_2_, mm Hg	25	67.1 (60.4, 75.6)	60.0 (51.8, 67.8)	69.7 (63.1, 76.5)	0.95 (0.93, 0.98)	< 0.001
HCO_3_−, mmol/L	25	23.0 (21.0, 25.0)	22.2 (20.0, 24.1)	23.0 (21.0, 25.0)	0.92 (0.83, 1.02)	0.117
SaO_2_, %	23	94.0 (91.8, 95.2)	91.4 (86.0, 94.0)	94.0 (92.8, 95.4)	0.85 (0.79, 0.92)	< 0.001
PaO_2_/FiO_2_ ratio, mm Hg	27	313 (271, 354)	262 (227, 300)	326 (290, 360)	0.99 (0.98, 0.99)	< 0.001
SaO_2_/FiO_2_ ratio	24	444 (421, 452)	423 (383, 441)	447 (433, 452)	0.99 (0.99, 1.00)	0.001

Data are n (%) or median (IQR). 95%CI, 95% Confidence Interval.

Severe course was death from any cause, use of mechanic invasive ventilation, or intensive care unit stay.

### Multivariate prediction models

**Figure 2:**
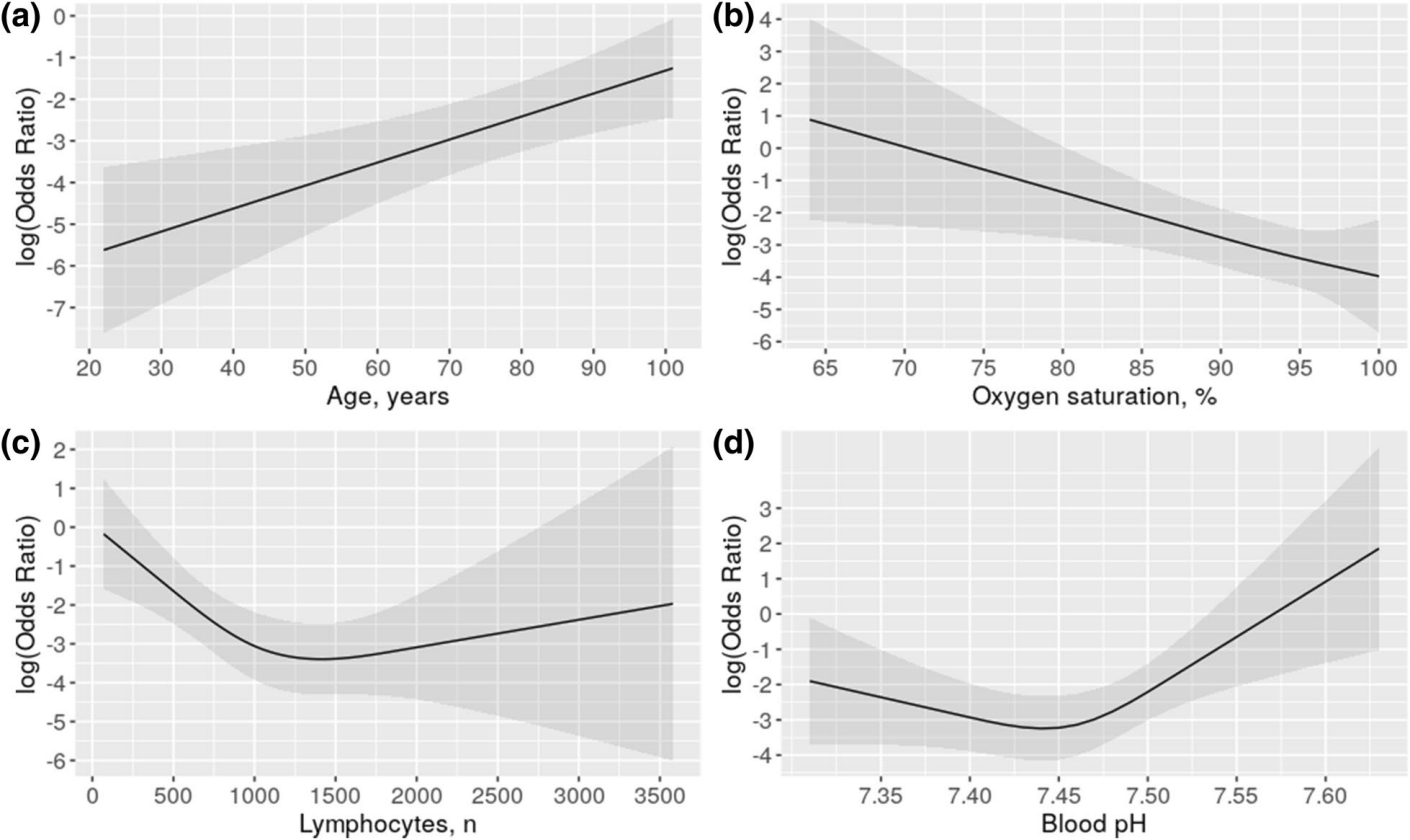
Effects of age, oxygen saturation, lymphocytes and pH on the risk for progression to severe disease (death, use of mechanic invasive ventilation, or intensive care unit stay) in patients hospitalized with COVID-19 pneumonia.

A regression model was built based on the aforementioned clinical, laboratory, gasometric, and radiographic data to predict the risk of progression to severe/critical disease. Five predictors were identified: Diabetes, Age, Lymphocyte count, SaO_2_, and pH (DALSH score, Nagelkerke R^2^ 0.45, Table 4). As shown in Fig. 2, whereas risk increases linearly with increasing age and decreasing SaO_2_, the relationship with leukocyte count and pH is not linear.

**Table 4:** Multivariate logistic regression analysis predicting severity in subjects hospitalized with COVID-19 pneumonia.

	β	SE(β)	OR (95%CI)	*p* value
**Intercept**	87.4549			
**Diabetes, yes**	1.4963	0.4480	4.46 (1.86, 10.74)	0.0008
**Age, yr**	0.0508	0.0171	1.05 (1.02, 1.09)	0.0030
**Lymphocyte count**				
rcs (1)	− 0.0036	0.0011	see Fig.2	0.0011
rcs (1ʹ)	0.0038	0.0017		0.0017
**SaO**_**2**_**, %**	− 0.1233	0.0434	0.88 (0.81, 0.96)	0.0046
**pH**				
rcs (1)	− 10.6378	7.7831	see Fig. 2	0.1717
rcs (1ʹ)	22.0366	9.5570		0.0212
AUC 0.87	R^2^ 0.44		Brier score 0.11	
AUC corrected 0.85	R^2^ corrected 0.38		Brier score corrected 0.13	

β indicates coefficient; SE, standard error; 95%CI, 95% confidence interval; rcs, restricted cubic splines (to interpret, see Fig. 2); AUC, Area under the ROC curve.

Severe course was death from any cause, use of mechanic invasive ventilation, or intensive care unit stay.

**Figure 3:**
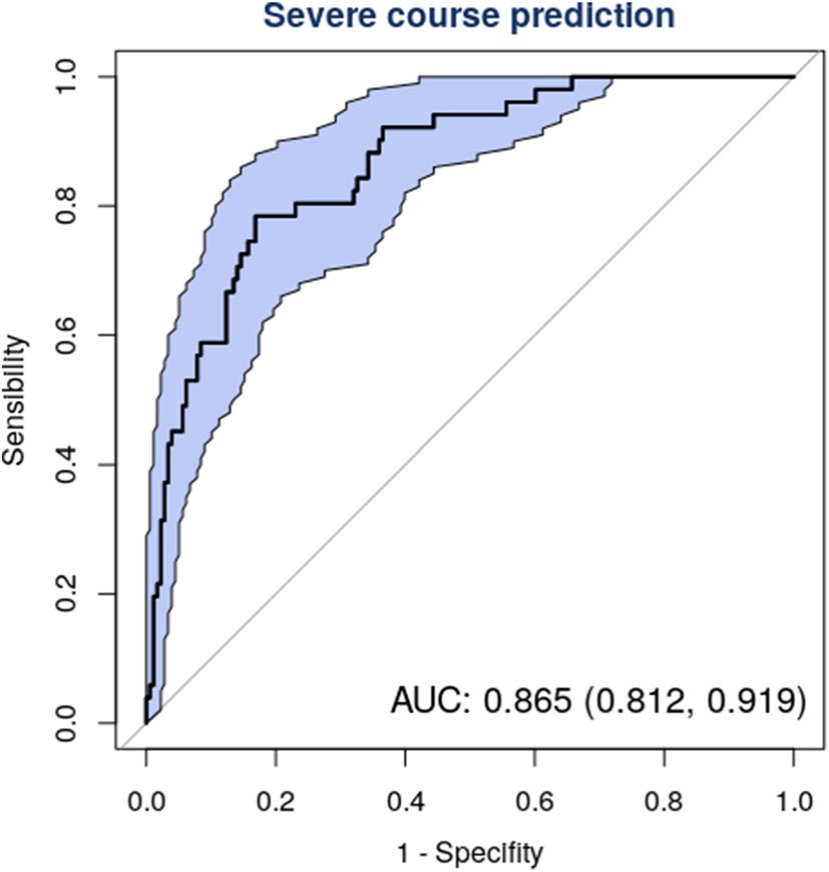
Receiver operating characteristic (ROC) curve for risk of severity (death, use of mechanic invasive ventilation, or intensive care unit stay) in COVID-19 patients with pneumonia. Figures show Area under the Curve [AUC (95% Confidence Interval)].

The receiver operating characteristic curve for this combination of predictors (Fig. 3) confirmed its good clinical performance (AUC 0.87 CI 0.81, 0.92). The AUC was higher than that obtained with the CURB-65 (AUC 0.73 CI 0.68, 0.78). The DALSH score showed an acceptable calibration (Brier score = 0.11, see supplementary material, Figure 1S). After correcting optimism by bootstrapping, Nagelkerke R^2^, AUC, and Brier score were 0.38, 0.85, and 0.13, respectively.

Figure 2S (see supplementary material) illustrates a method to estimate the risk of progression to severe disease based on an overall score calculated by the sum of the individual scores obtained in the five variables of the model.

Table 5 shows the individual score of each of the DALSH predictors. The total score indicates the estimated individual risk of severity of each patient. For instance, a patient with diabetes, 31 points; 70 years old, 50 points; and SaO_2_ 90%, 19 points; has a total of 100 points which is equivalent to the risk about 23%. The same patient with a lymphocyte count of 1000 cells/mm^3^, 10 points; has a total of 110 points which is equivalent to a risk of 32–33%. (Table 5, Figure S2). As many as 30% (n = 69) of patients had a ≤ 5% risk of progression (arbitrarily considered as low risk); 39% (n = 89) had a risk of 6–25% (intermediate risk), and 31% (n = 71) had a > 25% risk (high risk). No patients with a DALSH score < 66 points (low risk) progressed to severe disease (ICU admission, need for invasive mechanical ventilation, or death); whereas 12% of patients with a DALSH score of 66–100 points (intermediate risk) progressed to severe disease; finally, 50% of patients with a DALSH score > 100 points progressed to severe disease. Half of patients identified as high-risk had a mean age of 74 years. Notably, low-risk patients had a mean age of 54 years and none had diabetes.

**Table 5:** The DALSH score calculation for predicting risk of severity in patients hospitalized with COVID-19 pneumonia.

DM	Points	Age, yr	Points	Lymphocytes, n	Points	SaO_2_, %	Points	pH	Points	Total points	Severity risk	Levels
No	0	20	0	150	62	60	100	7.30	24	18	0.005	Low
Yes	31	30	10	250	55	65	86	7.35	15	32	0.010	
		40	20	500	39	70	73	7.40	5	66	0.050	
		50	30	750	23	75	59	7.45	0	81	0.100	Medium
		60	40	1000	10	80	46	7.50	14	101	0.250	
		70	50	1500	0	85	32	7.55	37	125	0.500	High
		80	59	2000	1	90	19	7.60	60	142	0.700	
		90	69	3000	7	95	8	7.65	82	170	0.900	
		100	79	4000	13	100	0			185	0.950	
		110	89							218	0.990	

DALSH, **d**iabetes, **a**ge, **l**ymphocytes, **s**aturation, p**H**; DM, diabetes mellitus; yr, years.

Severe course was death from any cause, use of mechanic invasive ventilation, or intensive care unit stay.

## Discussion

Given the high transmission rate, potential severity of symptoms, and scarcity of resources that may result from the COVID-19 pandemic, predicting the course of coronavirus is crucial to guaranteeing that patients receive the best care possible. In this study we derived and validated a clinical prediction rule for the prognosis of hospitalized patients with COVID-19 pneumonia. Our results also show that the presence of diabetes, advanced age, lymphopenia, hypoxemia and pH alterations on admission were all associated with disease severity.

The course of COVID-19 pneumonia is uncertain and may lead to death. Predicting the course of COVID-19 pneumonia at 24 h of admission based on clinical data is challenging but crucial. In a large number of cases, the disease can be controlled by closely monitoring the patient, but severely-ill patients will need aggressive treatment and intensive care. Predicting the course of the disease will enable the early adoption of a management approach in line with the estimated prognosis.

A multiplicity of studies have demonstrated that age is a relevant predictor of progression and mortality^5,7,22,23^, which is confirmed by the results of this study (OR 1.06 CI 1.03, 1.09; p = 0.000). This may be explained by age-related effects on T- and B-cell function and the excessive production of type 2 cytokines. Immunosenescence, or age-related defects in the human immune system, affects the adaptive immune response, as evidenced by major defects in cell-mediated immunity and impairment of humoral immune responses with age. These alterations impair the body’s ability to control viral replication and prolonged inflammatory response, thereby resulting in disease progression^24^.

Pneumonia may induce ischemia, endothelial dysfunction, and alterations in atherosclerotic plaques, which increases the short-term risk of cardiovascular events, especially in patients with previously known cardiovascular disease^25^. This may explain the fact that some studies—but not all^1^—have revealed a relationship between previous cardiovascular disease and a poorer prognosis of COVID-19^5,7,22^. In our study, patients with a previous diagnosis of heart failure were very likely to have a bad prognosis (OR 10.6 CI 3.25, 35.5; p < 0.001). Nevertheless, a relationship was not observed with ischemic heart disease. A variety of studies have established a relationship between diabetes and disease progression^5,7,22,23,26^. In our case, COVID-19 pneumonia was five-fold more likely to progress to severe disease in patients with diabetes (OR 5.20 CI 2.61, 10.35; p < 0.001). However, no studies have been conducted to date to clarify the relationship between diabetes and COVID-19.

Although prognosis is favourable in most patients, those with COVID-19 pneumonia can develop dyspnea and hypoxemia. The underlying pathophysiology of disease progression seems to be that of severe acute respiratory distress syndrome (ARDS). In these cases, the activation of alveolar macrophages by COVID-19 may trigger the release of powerful proinflammatory mediators and chemokines, the accumulation of neutrophils and monocytes, and the production of toxic mediators, which would lead to a loss of alveolar endothelial and epithelial barrier function, and ultimately induce alveolar and interstitial edema^27^. Laboratory test values alterations identified in critical patients with COVID-19 pneumonia could be associated with adverse ARDS outcomes. This suggests that infection could induce cell-mediated immunity alterations, the activation of coagulation, and myocardial, hepatic or renal damage. In line with other publications, our study demonstrates that the risk of disease progression increases with alterations in SaO_2_^7^ (OR 0.85 CI 0.79, 0.92; p < 0.001); LDH^5–7,9,22^ (OR 1.23 CI 1.05, 1.44; p = 0.012); CRP^6,7,9^ (OR 1.12 CI 1.06, 1.18; p < 0.001); IL-6^6^ (OR 1.01 CI 1.01, 1.02; p < 0.001); and lymphocyte count^5–7,9,22^ (OR 0.84 CI 0.77, 0.91; p < 0.001), which are the most commonly used inflammation markers.

Our prognostic model is based on five predictors: diabetes, age, lymphocyte count, SaO_2_ and pH, which we call the DALSH score. This score has demonstrated a good discrimination power (AUC 0.87 CI 0.81, 0.92). The DALSH score may be highly useful in clinical practice as these predictors can be easily determined in most settings and are usually recorded on admission. In addition, this score makes it possible to establish risk levels (even arbitrarily: ≤ 5%, low; 6–25%, intermediate and > 25% high) that may be useful to guide decision-making. Thus, a patient identified as low-risk is unlikely to experience disease progression; a relatively low number of patients identified as intermediate-risk will develop disease progression, and a large proportion of high-risk patients will experience disease progression. The cases observed in our series were 0%, 12% and 50%, respectively. Determination of risk would help clinicians adopt the appropriate therapeutic measures. However the non-linear relationships between two predictors (lymphocyte count and pH) and the outcomes means that its use in clinical settings is not so simple.

Any pH alteration is associated with a higher risk for progression. pH decrease is probably related to hypercapnia secondary to alveolar hypoventilation, whereas its increase may be due to the presence of respiratory alkalosis with progressive hyperventilation caused by hypoxemia. The two settings can co-occur with disease progression (Fig. 2D). A decrease in lymphocytes count causes an increase in OR, and the same occurs when it increases, although the effect is more subtle (Fig. 2C). A recent systematic literature review on prognostic models for predicting mortality or progression to severe disease revealed that these models have good to excellent performance. However, only a study involved patients from countries other than China, and all studies had been categorized as being at a high risk of bias, mainly because the sample of control patients was not representative, patients who had not experienced the event of interest by the end of the study were excluded; and model overfitting. The performance estimated of these studies were rated to be optimistic and misleading^11^. This systematic review includes 107 studies describing 145 prediction models for identifying people at risk in the general population; diagnostic models for detecting COVID-19; and prognostic models for predicting mortality risk, progression to severe disease, intensive care unit admission, ventilation, intubation, or length of hospital stay. The most frequently reported predictors of diagnosis and prognosis of COVID-19 are age, body temperature, lymphocyte count, and lung imaging features, similar to those found in our study. Unlike some of those studies, in our study all patients were followed-up until discharge or death, and objective predictors were used. A major strength of our study is the high-quality data obtained for all predictors and the minimal rate of missing data.

This study has several limitations. First, since the model was developed in a single population, a major limitation is the lack of external validation. Second, sample size was not large enough to adequately develop a multivariate regression model in which 53 predictors were entered. For this reason, after multivariate analysis, we resampled the development sample using a bootstrapping technique. The purpose was to avoid overfitting and estimate the stability of the dataset. As a result, five variables were retained in the model.

In summary, our study identified five straightforward, objective predictors easily determined on admission, which are associated with progression to severe or critical state in hospitalized patients with COVID-19 pneumonia. A risk score based on these factors predicted disease progression and allowed us to adopt therapeutic measures in accordance with patient’s prognosis from the very moment of diagnosis. To verify the external validity of the DALSH score, studies from the other hospitals even in the same area, and also from the other areas, should be required as this study was conducted using the patients only from one hospital.

## Supplementary information

Supplementary Figure Legends.

Supplementary Figure S1.

Supplementary Figure S2.
