# Supplementary material for: Development and validation of a clinical score to estimate progression to severe or critical state in COVID-19 pneumonia hospitalized patients
Source: Sci Rep. 2020 Nov 13;10:19794. doi: 10.1038/s41598-020-75651-z (PMC7666132; doi:10.1038/s41598-020-75651-z)
Supplement: Supplementary file 1 — Supplementary Figure Legends. [file 41598_2020_75651_MOESM1_ESM.docx]

**SUPPLEMENTARY INFORMATION**

**FIGURE LEGENDS**

**Figure S1.** Calibration plot for the final model in imputed validation samples. Observed severity disease vs. predicted severity disease. Diagonal line indicates perfect calibration.

**Figure S2**. Nomogram for prediction to severe disease among COVID-19 patients with pneumonia.

***Instructions:*** Locate the patient's age on the (Age) axis. Draw a line straight upward to the point's axis to determine how many points toward the probability of progression to severe disease the patient receives for his/her Age. Repeat the process for each variable. Sum the points achieved for each of the predictors. Locate the final sum on the Total Points axis. Draw a line straight down to find the patient's probability of having severe disease.

DM = Diabetes Mellitus.
