# Supplementary figures and images for: Development and validation of a clinical score to estimate progression to severe or critical state in COVID-19 pneumonia hospitalized patients
Source: Sci Rep. 2020 Nov 13;10:19794. doi: 10.1038/s41598-020-75651-z (PMC7666132; doi:10.1038/s41598-020-75651-z)

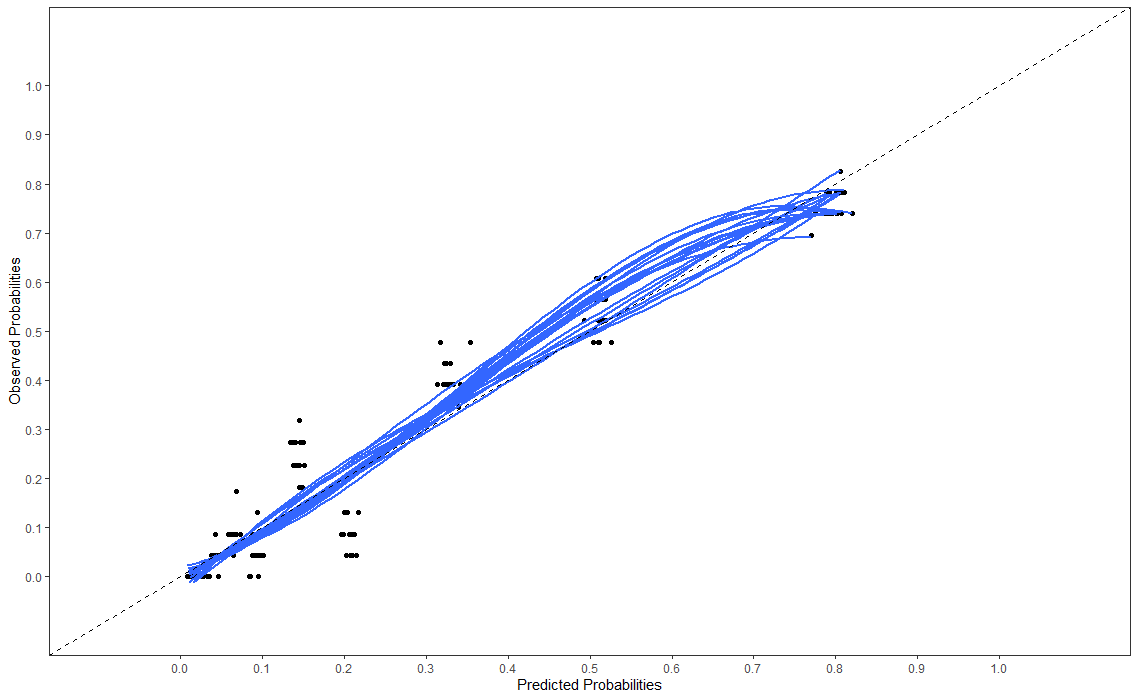

Supplement: Supplementary file 2 — Supplementary Figure S1. [file 41598_2020_75651_MOESM2_ESM.tiff]

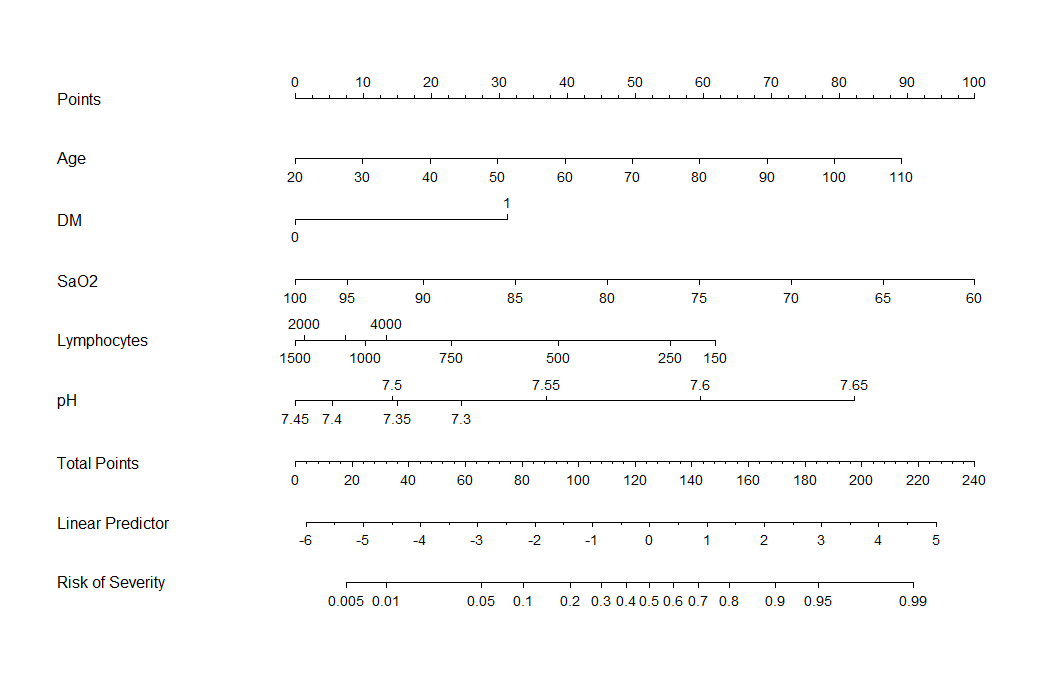

Supplement: Supplementary file 3 — Supplementary Figure S2. [file 41598_2020_75651_MOESM3_ESM.tiff]
